# Toscana virus encephalitis in Southwest Germany: a retrospective study

**DOI:** 10.1186/s12883-021-02528-7

**Published:** 2021-12-22

**Authors:** R. Dersch, A. Sophocleous, D. Cadar, P. Emmerich, J. Schmidt-Chanasit, S. Rauer

**Affiliations:** 1 Clinic of Neurology and Neurophysiology, Medical Center – University of Freiburg, Faculty of Medicine, University of Freiburg, 79106 Freiburg, Germany; 2 WHO Collaborating Centre for Arbovirus and Haemorrhagic Fever Reference and Research, Bernhard Nocht Institute for Tropical Medicine, 20359 Hamburg, Germany; 3 Faculty of Mathematics, Informatics and Natural Sciences, Universität Hamburg, 20148 Hamburg, Germany

**Keywords:** Emerging viruses, Encephalitis, Meningitis, Sandfly fever, Toscana virus, Neuroinfectious diseases

## Abstract

**Background:**

Toscana virus (TOSV) is an arthropod-borne virus transmitted by phlebotomine sandflies (*Phlebotomus* sp.) widespread throughout the Mediterranean having the potential to cause meningoencephalitis in humans. In Germany, the vectors of TOSV are introduced recently and become endemic especially in Southwestern Germany. As TOSV is not investigated regularly in patients with meningoencephalitis, cases of TOSV-neuroinvasive disease may remain mostly undetected.

**Methods:**

We conducted a retrospective cohort study on patients with meningoencephalitis without identification of a causal pathogen from 2006 to 2016. Serologic assessment for anti-TOSV-IgG and IgM was performed on serum and CSF. Demographic, clinical and CSF data from TOSV-positive patients were compared to a cohort of patients with meningoencephalitis due to enterovirus. Informed consent was obtained from all included patients.

**Results:**

We found 138 patients with meningoencephalitis without identified causal pathogen. From 98 of these patients CSF and serum was available for further testing. Additionally, we included 27 patients with meningoencephalitis due to enterovirus. We identified two patients with serological confirmed TOSV-neuroinvasive disease (TOSV-IgM and IgG positive, 2%) and two patients with possible TOSV-neuroinvasive disease (isolated TOSV-IgM positive, 2%). Overall, TOSV-neuroinvasive was detected in 4% of our cases with suspected viral meningoencephalitis. None of them had a history of recent travel to an endemic area.

**Conclusions:**

We found cases of TOSV-neuroinvasive disease in our German cohort of patients with meningoencephalitis. As no recent history of travel to an endemic area was reported, it remains probable that these cases resemble autochthonous infections, albeit we cannot draw conclusions regarding the origin of the respective vectors. TOSV could be considered in patients with meningoencephalitis in Germany.

## Background

Viral meningoencephalitis is suspected in patients with altered mental status, headache, fever and cerebrospinal fluid (CSF) pleocytosis often accompanied by neurological deficits, seizures and alterations in cerebral imaging or electroencephalography (EEG). The most common viral pathogens causing meningoencephalitis in central Europe are considered the herpes simplex virus, varicella-zoster virus, enteroviruses and tick-borne encephalitis virus [1]. There are considerable regional differences, especially regarding arthropod-borne transmission, which relates closely to the habitat of the respective arthropod vector.

However, in patients with suspected viral meningoencephalitis the causative pathogen cannot be identified in approximately 50% [2]. In the Mediterranean area, Toscana virus (TOSV) is one for the most prevalent viruses in patients with meningoencephalitis [3]. Toscana virus is an arthropod-borne enveloped, negative-stranded RNA virus transmitted by sandflies (*Phlebotomus* sp.) and belongs to the *Phlebovirus* genus within the *Phenuiviridae* family. In humans, infections with TOSV are usually asymptomatic or mild, but can also cause neuroinvasive disease called sandfly fever. In Central Italy, TOSV is responsible for approximately 80% of the cases of aseptic meningitis during the summer [4]. The incubation period is usually 3–7 days, but can be as long as 2 weeks. Typical symptoms are headache, fever, neck rigidity, myalgia, photophobia and focal neurological signs [3]. In most cases, symptoms resolve after 7–10 days of febrile illness. However, severe courses of disease and cases of fatal encephalitis have been reported previously [5, 6]. In cases of acute TOSV-infection, usually -TOSV-IgM and -IgG are detectable in serum. IgM decreases over time and cease to be detectable whereas IgG can be detected years after contact with TOSV [7].

In Germany, cases of sandfly fever caused by TOSV are rare and usually occur in travelers after exposure in endemic areas [5, 8]. However, sandflies from the *Phlebotomus* species are increasingly detected in areas where these vector have not been reported previously, especially in Southwest Germany in the Upper Rhine Valley [9]. Sandfly populations can now be found over several years at the same site [9]. Thus, autochthonous populations can be assumed. Climate change with an elevation of the annual mean temperature over the last decades is discussed as a relevant factor for the expansion of the habitat of sand flies [9].

As competent vectors of TOSV are present in southwestern Germany, an elevated risk of transmission of this virus to humans seems possible. As awareness of TOSV is currently low, TOSV is usually not incorporated in the diagnostic work-up of patients with viral meningoencephalitis in Germany. As a causal pathogen cannot be identified in approximately 50% of cases with suspected viral encephalitis, it is possible that cases of TOSV-neuroinvasive disease remain undetected. To identify possible undetected cases of TOSV-neuroinvasive disease, we performed a retrospective cohort study on patients who were diagnosed as having a suspected viral meningoencephalitis without identified pathogen. We serologically tested CSF and serum for TOSV IgM and IgG. As TOSV-cases are undistinguishable from other viral pathogens regarding clinical features, we investigated whether there are specific CSF profiles or laboratory test results that could guide diagnostic assessment in cases of viral meningoencephalitis in the future.

## Methods

We searched medical records of a tertiary care center in Southwest Germany (Clinic of Neurology and Neurophysiology, Medical Center – University of Freiburg) from January 2006 – December 2016 for patients with suspected viral meningoencephalitis (ICD 10-code A87). For being considered as having possible viral meningoencephalitis, patients had to exhibit headache, altered mental state, fever and CSF pleocytosis. Patients were excluded if a causal pathogen was identified (diagnostic workup includes PCR for herpes simplex virus, varicella zoster virus, Enterovirus, eubacterial PCR, PCR for *Listeria* as well as serology for *B. burgdorferi*, syphilis and tick-borne-encephalitis virus) or onconeural antibodies (Hu, Yo, Ri, CV2/CRMP5, Amphiphysin, Ma1, Ma2, SOX1, GAD65, Tr (DNER), Zic4) or antibodies against neuronal cell-surface antigens (NMDAR, LGI1, AMPAR, CASPR2, GABAR) were detected. To investigate clinical spectrum and the profile of changes in CSF, data on clinical course and results from laboratory testing as well as CSF analysis were collected. A control group was compiled of patients with aseptic meningoencephalitis due to infection with enterovirus confirmed with PCR. After initial diagnostic workup, CSF and serum was stored for further investigation at − 80 °C. CSF and serum samples were stored, treated, and analyzed in the same manner for all included patients. Written informed consent was obtained from each patient. The study was conducted according to Helsinki criteria and received approval by the local ethics committee of the Medical Center – University of Freiburg, Faculty of Medicine (EK FR 536/19). Demographical data and information on initial symptoms, treatment and course of disease as well as data on CSF analysis and laboratory analyses were obtained from the medical records.

Serological testing for anti-TOSV-IgG and IgM and PCR for Phlebovirus-RNA was performed at the Bernhard-Nocht-Institute for Tropical Medicine, Hamburg, Germany [10]. Immunofluorescence assays (IFA) for various sandfly-borne phleboviruses (TOSV, SFNV and SFSV) were performed with virus-infected Vero E6 cells. In brief, Vero cells were spread onto slides, air dried, and fixed in acetone. Serum samples were serially diluted in phosphate-buffered saline (PBS) starting with an initial dilution of 1:10, added to the cells, and incubated for 90 min at 37 °C. After washing with PBS, slides were incubated with Fluoresceine isothiocyanate-labeled rabbit anti-human IgG and IgM antibodies (SIFIN, Berlin, Germany) at 37 °C for 25 min. IgG titers or IgM titers of 1:20 or more were considered positive.

All samples were analyzed in triplets. For a diagnosis of definite/confirmed TOSV-infection evidence of anti-TOSV-IgM and -IgG was required [7]. Cases with isolated anti-TOSV IgM antibodies were regarded as possible TOSV-infections. Cases with isolated anti-TOSV IgG antibodies most likely represent residual antibody titers after a TOSV-infection in the past with no relation to the acute presentation of the patient.

## Results

We found 138 patients with suspected viral meningoencephalitis without identified causal pathogen. Of these patients, serum or CSF was available from 98 patients for further testing, 40 patients without available specimen were excluded from further analysis. In the control group of patients with meningoencephalitis due to enterovirus we included 27 patients. In the group of patients with suspected viral meningoencephalitis of unknown origin we could identified 2 cases with definite TOSV-infection (anti-TOSV-IgM and IgG TOSV positive), 2 cases with possible TOSV-infection (isolated anti-TOSV-IgM positive) and 2 cases with residual antibody titers (isolated anti-TOSV-IgG positive). One patient with definite TOSV-infection also showed positive anti-TOSV-IgG in CSF. In the two patients with both anti-TOSV-IgM and anti-TOSV-IgG antibodies follow up testing 1–2 weeks after baseline showed increasing titers for both IgM and IgG. Unfortunately, in patients with only anti-TOSV-IgM antibodies no material was available for follow-up serology. PCR for RNA of Phleboviruses was negative in all samples. Demographic and clinical data for the included patients are shown in Table 1. None of the TOSV-patients had a history of travel to regions with known endemic TOSV-infection.

**Table 1:** Demographical and clinical characteristics and results from CSF analysis of included patients. For groups, values resemble mean with SD in brackets

	Case 1	Case 2	Case 3	Case 4	TOSV overall (***n*** = 4)	Enterovirus (***n*** = 27)
**TOSV-Antibiodies in serum (baseline)**	IgM: 1/320 IgG: 1/1280	IgM: 1/1280 IgG: 1/320	IgM: 1/160	IgM: 1/80		
**TOSV-Antibiodies in serum (1–2 weeks)**	IgM: 1/1280 IgG: 1/5120	IgM: 1/640 IgG: 1/640	n.a.	n.a.		
**TOSV-Antibodies in CSF**	IgG (1:20)	n.a.	negative	negative		
**TOSV diagnosis**	definite	definite	possible	possible		
**Age [yrs]**	70	21	71	80	60.5 (26.7)	35
**Sex**	m	f	f	f		14 f: 13 m
**Clinical presentation**	meningoencephalitis	meningitis, myelitis	meningoencephalitis	meningoencephalitis		meningitis (100%)
**CSF cell count [/μl]**	162	681	10	53	226.5 (309.7)	319.8 (395.6)
**Total protein [mg/dl]**	736	3460	612	498	1326.5 (1425.7)	60.7 (21.9)
**AQ**^**a**^	9.4	52.3	2.67	7.9	18.1 (23.0)	7.2 (2.8)
**Intrathecal Immunoglobulin-synthesis**	IgM	none	none	none		IgM *n* = 2 IgA *n* = 2
**Lactate [mmol/l]**	3.62	4.93	2.67	1.91	3.3 (1.3)	2.2 (0.6)
**CSF cytology**	lymphocytes 90%, monocytes 10% activated lymphocytes +	granulocytes 60% lymphocytes 30% monocytes 10%	lymphocytes 90% monocytes 10%	lymphocytes: 90% monocytes: 10%		
**MRI/CT abnormalities**	none	none	none	none	none	none
**WBC**^**a**^	5.52	6.6	9.0	7.8	7.23 (1.5)	8.1 (2.5)
**CRP [mg/l]**^**a**^	< 3	8	9	53	23.3 (25.7)	13.7 (14.1)
**Transaminases**^**a**^	normal	AST, ALT, GGT elevated	normal	normal		1/27 elevated GGT/ALT
**Lenght of stay in hospital [days]**	7	18	14	16	13.75 (4.8)	5.5 (2.8)
**Clinical outcome**	no sequelae	Residual paraparesis, slight cognitive impairment	no sequelae	no sequelae	1/4 (25%) sequelae	3/27 (11%) sequelae (headache *n* = 1, fatigue *n* = 2)

^**a**^*AQ* albumin quotient, *WBC* white blood cell count, *CRP* C-reactive protein, *AST* aspartat transaminase, *ALT* alanin transaminase, *GGT* gamma-glutamyltransferase

One patient (case 2) had a severe affection of the CNS with myelitis and prolonged recovery requiring inpatient rehabilitation treatment. No specific imaging abnormalities were found in patients with TOSV-neuroinvasive disease.

Due to the small sample size of patients with TOSV-neuroinvasive disease, statistical comparisons with patients with meningoencephalitis due to enterovirus were not deemed justified.

## Discussion

In our cohort of 98 patients with suspected viral meningoencephalitis with unknown origin we found 2 cases of definite TOSV-neuroinvasive disease (2%) and 2 cases of possible TOSV-neuroinvasive disease (2%). Overall, TOSV antibodies were detected in 4% of our cases with suspected viral meningoencephalitis with unknown origin. One patient had severe affection of the CNS with myelitis, all other patients had a mild course of disease. The individual patients did not reported recent travel to regions with endemic TOSV-infection. Therefore, these cases are likely to represent autochthonous cases of TOSV-infection. In our retrospective study, we are unable to distinguish whether the infection was acquired from a local population of vectors or from migrating vectors (e.g. introduced in cars from travelers from endemic regions).

Due to the small sample size of patients with TOSV-neuroinvasive disease, statistical comparison with patients with meningoencephalitis due to enterovirus were not justified. However, regarding data from CSF analysis and laboratory analysis, no distinct pattern regarding TOSV-neuroinvasive disease emerges.

As our study was retrospective and had a descriptive design, several limitations have to be discussed. Patients were screened based on the diagnosis of suspected viral meningoencephalitis in the initial medical records. We may have missed eligible patients that were classified otherwise e.g. as fever of unknown origin or post-infectious headache.

Surplus CSF and serum samples were stored at − 80 °C, with potentially disadvantageous effects on sample quality. A prospective study with timely initiation of diagnostic procedures would be desirable to enhance quality of samples and enable further diagnostic procedures like PCR. Two of the three patients with possible TOSV-neuroinvasive disease had only evidence of anti-TOSV IgM. A follow-up investigation regarding seroconversion would have been informative but was unfortunately not possible as patients were unavailable for further evaluation. Whether these cases resemble true TOSV-infections or were due to cross-reacting antibodies remains unclear. Recently, we demonstrated that a bacterial-induced immunopathological state mimicked acute sandfly fever and led to unspecific low TOSV-IgM and -IgG titers in IFA [11]. However, low unspecific TOSV IFA titers were excluded in our study.

PCR for RNA of Phlebovirus was negative in all samples. However, as viremic period is short in individuals infected with TOSV, negative PCR is of limited use in diagnosing TOSV-neuroinvasive disease.

Other case series on patients with TOSV neuroinvasive disease report similar CSF findings and also a small proportion of patients with severe CNS involvement [12]. Some cases of hydrocephalus in the course if TOSV neurinvasive disease have been reported, however none of our patients had signs of hydrocephalus in cerebral imaging [13, 14]. Previous studies on CSF in TOSV-neuroinvasive disease showed moderate lymphocytic CSF pleocytosis and moderate elevation of CSF protein [12]. CSF characteristics in our patients with TOSV-neuroinvasive disease were similar to those previous reports, albeit these findings remain indistinguishable from other causes of viral meningoencephalitis. Case 2 showed the highest CSF pleocytosis and a mixed cell pleocytosis with high fraction of granulocytes. This may resemble the larger extend of neuroinflammation in this case with severe spinal involvement. Anti-TOSV-IgG in CSF were only detectable in one patient and negative in two patients. This may indicate that TOSV induces an acute systemic inflammation similar to immune responses seen in other neurozoonotic diseases, like tick-borne-encephalitis.

Our study shows that autochthonous cases of meningoencephalitis due to TOSV infection may exist in Germany. Information on distribution of viral pathogens may influence clinical decisions on diagnostic approaches when faced with patients with suspected viral meningoencephalitis. Dissemination of viral pathogens may also be relevant regarding measures of sandfly control, as such measures are already in place for other arthropod vectors of emerging viruses like Aedes spec [15].. Identification of patients with TOSV-neuroinvasive disease could have implications for public health, as specific vector control programs could be considered around a diagnosed patient.

Despite usually favorable courses of disease, severe cases with parenchymal involvement and residual neurological symptoms may occur.

Further prospective studies could generate more information on prevalence of TOSV-neuroinvasive disease in Germany, especially in areas at-risk at developing established populations of sand flies.

As currently no treatment or vaccine is available, no specific treatment can be offered to patients diagnosed with TOSV-neuroinvasive disease. However, identifying TOSV as the causal agent in a patient with meningoencephalitis can help to avoid unnecessary treatment with antibiotics and antiviral agents [16].

As prevalence is expected to rise, TOSV should be considered as a possible pathogen by clinicians in Germany when faced with patients with viral meningoencephalitis [9].

## Data Availability

The data that support the findings of this study are available on reasonable request from the corresponding author.
